# Deep Convolutional Neural Network-Based Brain Magnetic Resonance Imaging Applied in Glioma Diagnosis and Tumor Region Identification

**DOI:** 10.1155/2022/4938587

**Published:** 2022-05-24

**Authors:** Zhen Chen, Ning Li, Changtao Liu, Shiwei Yan

**Affiliations:** Department of Neurosurgery, The First People's Hospital of Lianyungang/The Affiliated Lianyungang Hospital of Xuzhou Medical University, Lianyungang 222000, Jiangsu, China

## Abstract

The aim of this study was to explore the application value of dynamic contrast-enhanced magnetic resonance imaging (DCE-MRI) based on a convolutional neural network (CNN) algorithm in glioma diagnosis and tumor segmentation. 66 patients with gliomas who were diagnosed and treated in the hospital were selected as the research objects. The patients were rolled into the high-grade glioma group (HGG, 46 cases) and the low-grade glioma group (LGG, 20 cases) according to the World Health Organization glioma grading standard. All patients received a conventional plain scan and a DCE-MRI. Parameters such as volume transfer constant (*K*^trans^), rate constant (*K*_*ep*_), extracellular volume (*V*_*e*_), and mean plasma volume (*V*_*p*_) were calculated, and the parameters of patients of each grade were analyzed. The efficacy of each parameter in diagnosing glioma was analyzed through a receiver operating characteristic curve. All images were segmented by the CNN algorithm. The CNN algorithm showed good performance in DCE-MRI image segmentation. The mean, standard deviation, kurtosis, and skewness of *K*^trans^ and *V*_*e*_, the standard deviation and skewness of *K*_*ep*_, and the mean and standard deviation of *V*_*p*_ were statistically considerable in differentiating HGG and LGG (*P* < 0.05). ROC analysis showed that the standard deviation of *K*^trans^ (0.885) had the highest diagnostic accuracy in distinguishing HGG and LGG. The values of *K*^trans^, *V*_*e*_, and *V*_*p*_ were positively correlated with Ki-67 (*r* = 0.346, *P* = 0.014; *r* = 0.335, *P* = 0.017; *r* = 0.323, *P* = 0.022). In summary, the CNN-based DCE-MRI technology had high application value in glioma diagnosis and tumor segmentation.

## 1. Introduction

Glioma is one of the most common primary tumors in clinical practice. Clinical statistics showed that it is responsible for 81% of central nervous system malignancies [1–3]. In general, tumors are classified into low grade (grades I and II) and high grade (grades III and IV) according to the degree of malignancy. Clinical data showed that more than half of patients with glioma have glioblastoma, the most malignant form of brain cancer. In recent years, surgery and radiotherapy and chemotherapy have made continuous progress, but clinical data showed that the survival time of patients with comprehensive treatment is still less than 15 months, and their prognosis is also one of the worst among all tumor patients [4–6]. Almost all gliomas develop into the highest-grade gliomas. Clinical data showed that the average time for grade II and III gliomas to progress to IV is five years and two years, respectively. The incidence and mortality of glioma are high. Relevant clinical studies showed that the annual incidence of glioma in China is 3–6/100,000, and the annual death rate is as high as 30,000. In recent years, the incidence of this disease is also showing an increasing trend year by year. Statistics showed that the incidence of glioma in China is increasing by 1.2%. This disease poses a huge threat to human life and health [7, 8].

Once a glioma is diagnosed, patients do not survive for more than two years. Therefore, the early diagnosis of glioma is very important for the treatment and prognosis of the disease. At present, glioma can be graded by detecting molecular markers of glioma [9]. However, these methods are not widely used because of their high cost, the need for a large amount of tumor tissue, and the subjective influence of staining reagents and doctors. And these tests are invasive and cannot be used again in living tissue, which is why they are not widely available. With the rapid development of imaging technology, diseases can be diagnosed by various imaging methods. Magnetic resonance imaging (MRI) is one of the most important methods for the diagnosis of glioma [10–12].

How to segment tumor regions from brain MRI images of patients has always been a key and difficult point in the clinical diagnosis of glioma. At present, the most important method for regional segmentation of tumors is still manual segmentation by doctors. Such a segmentation method not only requires doctors to master strong professional knowledge but also wastes a lot of time and increases the workload of doctors. Therefore, the study of an automatic segmentation method of glioma has great positive significance for the diagnosis and treatment of glioma. However, the infiltration of tumor cells into surrounding tissues makes the tumor boundary blurred [13]. In addition, affected by the imaging principle of the MRI image itself, the grayscale range of images obtained by the same patient under different conditions is different. These characteristics of glioma lead to increased difficulty in image segmentation, so the traditional image segmentation methods cannot achieve good segmentation results. In recent years, the deep convolutional neural network (CNN) algorithm has shown considerable advantages in computer vision compared with other traditional machine learning algorithms. When the CNN algorithm performs image segmentation, it directly takes the original image as input and does not need to manually extract features. It can directly extract representative features from the image [14]. At present, many scholars have applied the CNN algorithm to glioma image segmentation [15, 16]. However, its segmentation effect varies greatly for different patients. In general, the CNN algorithm has a high application prospect and value in the segmentation of glioma. However, there is still a long way to go before it can be put into clinical application [17].

In this research, patients with glioma were taken as the research object, and dynamic contrast-enhanced (DCE)-MRI under the CNN algorithm was used to diagnose and segment patients with tumors, and the differences between the DCE-MRI-related parameters of patients with glioma of different grades were discussed and analyzed, to provide a good reference and basis for the diagnosis and treatment of clinically related diseases.

## 2. Research Methods

### 2.1. Research Objects

From March 2019 to March 2020, 66 patients with gliomas in hospital were selected as the research objects, including 36 male patients and 30 female patients. The mean age of the patients was 53.6 ± 11.3 years. According to the World Health Organization (WHO) glioma grading standard, the patients were rolled into the high-grade glioma group (HGG, 46 cases) and the low-grade glioma group (LGG, 20 cases). All studies obtained patient informed consent and this study had been approved by the ethics committee of the hospital.

Inclusion criteria were as follows: patients diagnosed with glioma after case diagnostic screening; patients with complete imaging and follow-up data; and patients with complete follow-up records. Exclusion criteria were as follows: patients with other malignant tumors at the same time; patients with other serious underlying diseases or with dysfunction of important organs such as the heart, lung, liver, and kidney; those who died of diseases or accidents other than glioma; and those who suffered from claustrophobia.

### 2.2. Imaging Studies

All cases were scanned by 3.0TMR with an 8-channel phased-array head coil. All patients underwent a routine plain scan and a DCE-MRI before surgery. Specific scanning parameters of conventional sequences were T1WI (TR/TE: 400 ms/2.48 ms, FOV: 230 × 230 mm^2^, matrix: 320 × 256, and bandwidth: 360 Hz/Px) and T2WI (TR/TE: 5,090 ms/91 ms, FOV: 230 × 230 mm^2^, matrix: 320 × 320, and bandwidth: 203 Hz/Px), with a layer thickness of 5 mm. DCE-MRI used cross-sectional T1 gradient 3D sequence scanning, and three groups of Tl-Vibe plain scans were performed before the examination (TR/TE: 3.89/1.31 ms, layer thickness: 3mm, FOV: 230 × 230 mm^2^; matrix: 224 × 161; flip angles: 5°, 10°, and 15°). A dynamic enhanced examination was then performed, including a total of 40 acquisitions. After the third collection, the contrast agent was injected through the cubital vein at a rate of 2.0–4.0 mL/s and a total amount of 0.1 mmol/kg. All localization levels of the DCE-MRI examination were consistent. The flip angle of the dynamic acquisition sequence was 15°, and the other parameters were the same as the previous plain scan sequence.

### 2.3. Image Processing

**Figure 1:**
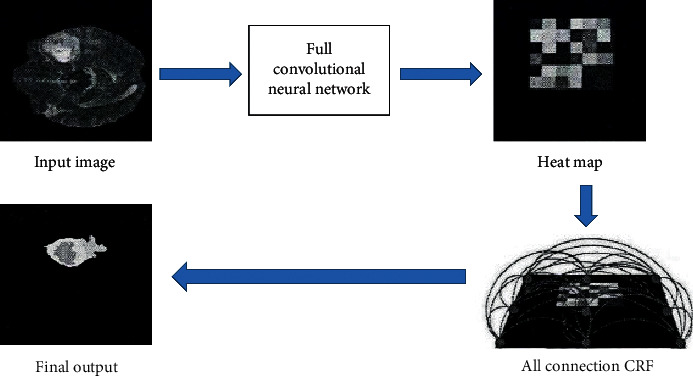
Image processing flow.

**Figure 2:**
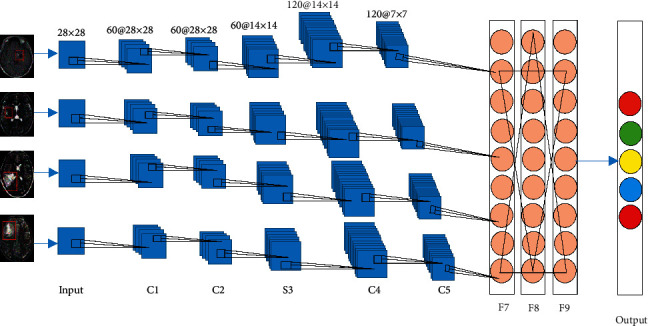
CNN model.

All MRI images were processed using the CNN algorithm. The specific image processing process includes the input image, CNN, heat map, CRF, output image, and other steps. The specific image processing process is shown in Figure 1. The CNN algorithm mainly includes a convolutional layer, a pooling layer, a fully connected layer, and a Softmax classification layer. The detailed CNN model is shown in Figure 2.

(1) $$\begin{array}{c} \overset{l}{\underset{j}{x}}=f\left(\sum_{i\in M_{j}}x_{j}^{l-1}•K_{ij}^{l}+b_{j}^{l}\right), \end{array}$$

where *l* is the number of layers, *K* is the convolution kernel, *x*_*j*_^*l*−1^ is the feature map output by the previous layer, *K*_*ij*_^*l*^ is the weight of the convolution kernel, *b* is the bias value, and *f*(•) is the activation function. The convolution operation has three modes of full convolution, same convolution, and valid convolution. The specific definitions are as follows.

(I) Full convolution
  (2) y=convx,w,′full'=y1,…,yt,…,yn+m−1∈Ryt=∑i=1mxt−i+1•wi t=1,2,…,n+m−1.
(II) Same convolution
  (3) y=convx,w,′same'=centerconvx,w,′full',n∈R.
(III) Valid convolution

(4) y=convx,w,′valid'=yl,…,yt,…,yn+m−1∈Ryt=∑i=1mxt+i−1wi t=1,2…,n+m−1.

The pooling layer can reduce the possibility of overfitting and improve the fault tolerance of the model. The calculation of the pooling layer is as follows:

(5) $$\begin{array}{c} x_{j}^{l}=f\left(\beta_{j}^{l}\text{down}\left(x_{j}^{l-1}\right)+b_{j}^{l}\right). \end{array}$$

*do*  *wn*(•) is the downsampling function, and *β* and *b* are the multiplicative bias and the additive bias, respectively. There are two common pooling operations in deep learning-based multifeature fusion classification algorithms, namely, average pooling and maximum pooling. The average pooling refers to taking the mean value within the filter range as the pooled output. The maximum value pooling refers to using the maximum value within the filter range as the pooling output.

As for the full connection process, in the multifeature fusion classification algorithm under deep learning, the full connection layer is a network node arranged linearly and encodes the output result of the previous layer into a one-dimensional vector. The fully connected layer is defined as follows:

(6) $$\begin{array}{c} x^{l}=f\left(w^{l}x^{l-1}+b^{l}\right). \end{array}$$

In the above equation, *w*^*l*^ is the network weight coefficient, *x*^*l*−1^ is the output feature map of the previous layer, and *b*^*l*^ is the fully connected layer bias item.

Softmax classification layer is a multiclassifier connected to the fully connected layer, which can complete more than two classification tasks and convert multiple outputs into probability values in the (0,1) interval. In logistic regression, the training set is *T*={(*x*^(1)^, *y*^(1)^),…, (*x*^(*m*)^, *y*^(*m*)^)}, the input sample is *x*^*i*^ ∈ *R*^*n*^, and *y*^(*i*)^ is the sample label. Then, it is assumed that the function (hypothesis function) is defined as follows:

(7) $$\begin{array}{c} h_{\theta }\left(x\right)=\frac{1}{\left(1+e^{-\theta_{X}^{T}}\right)}. \end{array}$$

The cost function *J*(*θ*) is minimized as follows:

(8) $$\begin{array}{c} J\left(\theta \right)=-\frac{1}{m}\left[\sum_{i=1}^{m}y^{\left(i\right)}\log\;h_{\theta }\left(x^{\left(i\right)}\right)+\left(1-y^{\left(i\right)}\right)\log\left(1-h_{\theta }\left(x^{\left(i\right)}\right)\right)\right]. \end{array}$$

The calculation of Softmax is as follows:

(9) $$\begin{array}{c} h_{\left(\theta \right)}\left(x^{\left(i\right)}\right)=\left[\begin{array}{c} p\left(y^{\left(i\right)}=1|x^{\left(i\right)},\theta \right)\\ p\left(y^{\left(i\right)}=2|x^{\left(i\right)},\theta \right)\\ \cdots \\ p\left(y^{\left(i\right)}=k|x^{\left(i\right)},\theta \right) \end{array}\right]=\frac{1}{\sum_{j=1}^{k}e^{\theta_{j}^{T}x^{\left(i\right)}}}\left[\begin{array}{c} e^{\theta_{1}^{T}x^{\left(i\right)}}\\ e^{\theta_{2}^{T}X^{\left(i\right)}}\\ \cdots \\ e^{\theta_{K}^{T}x^{\left(i\right)}} \end{array}\right]. \end{array}$$

Learning on the training sample *T* minimizes the damage function of Softmax. The expression of the minimum loss function is as follows:

(10) $$\begin{array}{c} J\left(\theta \right)=-\frac{1}{m}=\left[\sum_{i=1}^{m}\sum_{j=1}^{k}1\left\{y^{\left(i\right)}=j\right\}\log\frac{e^{\theta_{i}^{T}x^{\left(i\right)}}}{\sum_{i=1}^{k}e^{\theta_{i}^{T}x^{\left(i\right)}}}\right], \end{array}$$

where 1{*y*^(*i*)^=*j*} indicates that if *y*=*j*, the value is 1; otherwise, it is 0; that is, the smaller the loss function, the closer the expected target.

The mean square error (MSE), peak signal-to-noise ratio (PSNR), and structural similarity (SSIM) are used to evaluate the segmentation effect quantitatively. The specific calculation methods of the three indicators are as follows:

(11) $$\begin{array}{c} \text{MSE}=\frac{1}{mn}{\sum_{i=0}^{m-1}\sum_{j=0}^{n-1}\left[I\left(i,j\right)-K\left(i.j\right)\right]}^{2},\\ \text{PSNR}=10\cdot \log_{10}\left(\frac{{\text{MAX}}_{I}^{2}}{\text{MSE}}\right)=20\cdot \log_{10}\left(\frac{{\text{MAX}}_{I}}{\sqrt{\text{MSE}}}\right),\\ \text{SSIM}\left(x,y\right)=\frac{\left(2\mu_{x}\mu_{y}+c_{1}\right)\left(2\sigma_{xy}+c_{2}\right)}{\left(\mu_{x}^{2}\right)\left(+\mu_{y}^{2}+c_{1}\right)\left(\sigma_{x}^{2}+\sigma_{y}^{2}+c_{2}\right)}. \end{array}$$

### 2.4. Pathological Specimen Analysis

All patients' tumors were surgically removed, then the tumor specimens were analyzed, and the gliomas were classified into grades I to IV. Ki-67 immunohistochemical staining was performed on the obtained specimen, the whole specimen was browsed, and then the area with the highest positive expression density was selected. 1,000 tumor cells were counted under a 200× microscope, and the positive percentage of tumor cells was taken as the highest Ki-67 labeling index. The specific staining methods of Ki-67 were as follows: I. After samples were taken, the tissues were fixed with neutral formaldehyde for 24–48 hours, followed by conventional paraffin embedding treatment. II. The sample was sliced to 5∼7 *μ*m. III. The samples were put into 10 mM citric acid buffer and microwaved for 10 minutes. IV. The slices were cooled at room temperature for 20 minutes. V. Steps III and IV were repeated. VI. Slices were cooled at room temperature for 20 minutes before being removed from citric acid, washed twice with Tris-HCl buffer, and then left in Tris-HCl for staining. VII. Finally, Ki-67 labeling was carried out according to the conventional method.

### 2.5. Statistical Methods

Statistical analysis of all data relied on SPSS 11.0 to complete. Measurement data were expressed as the mean ± standard deviation ($\bar{x}$ ±*s*), and *t*-test was used to test the significance of patient data before and after surgery. The count data were expressed as actual number and percentage, and the significance test was carried out by *χ*^2^ test. *P* < 0.05 was considered statistically considerable.

## 3. Results

### 3.1. Typical Case Image Display

**Figure 3:**
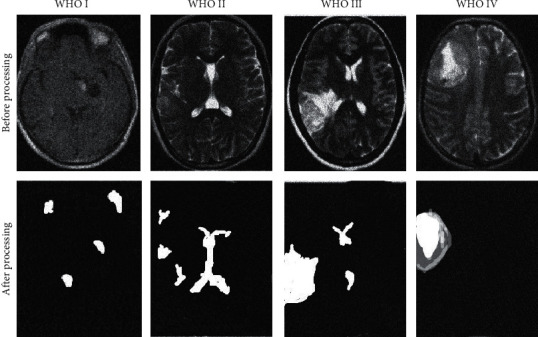
Typical case images.

The typical case images are shown in Figure 3. Analysis of Figure 3 shows that MRI can distinguish different grades of gliomas well. The CNN algorithm can segment lesions from MRI images of glioma patients more accurately.

### 3.2. Comparison of Histogram Parameters of *K*^trans^, *K*_*ep*_, *V*_*e*_, and *V*_*p*_ Values in HGG and LGG

**Figure 4:**
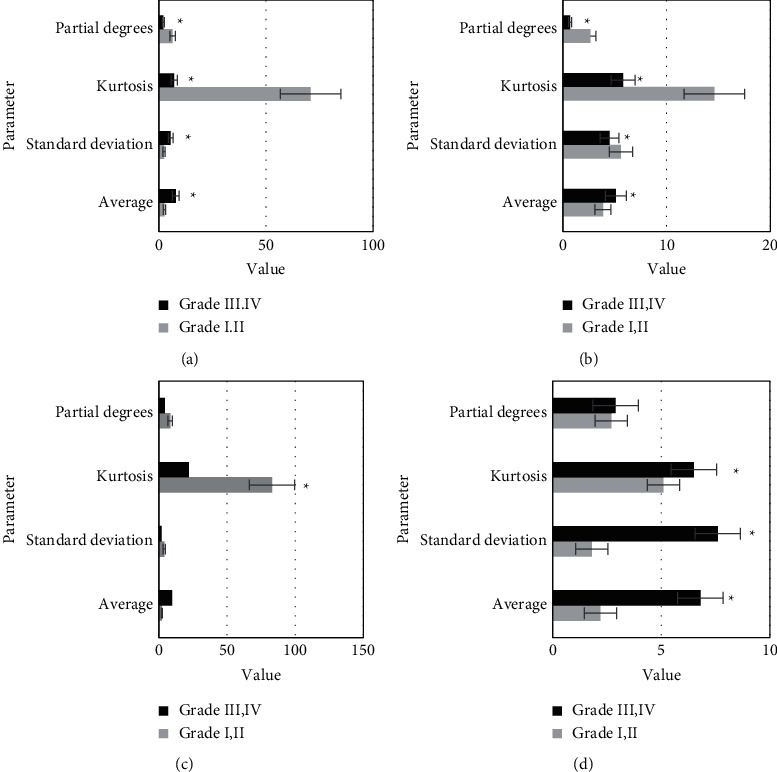
Comparison of histogram parameters of *K*^trans^, *K*_*ep*_, *V*_*e*_, and *V*_*p*_ in HGG and LGG. (a) K^trans^; (b) K_*ep*_; (c) *V*_*e*_; (d) *V*_*p*_ compared with the LGG group,  ^*∗*^*P* < 0.05.

The comparison results of the histogram parameters of *K*^trans^, *K*_*ep*_, *V*_*e*_, and *V*_*p*_ values of HGG and LGG are shown in Figure 4. Figure 4 shows that there were significant differences in mean value, standard deviation, kurtosis, and skewness of *K*^trans^ between the HGG group and the LGG group (*P* < 0.05). The mean value, kurtosis, and skewness of *K*_*ep*_ were significantly different between the two groups, *P* < 0.05. The mean value, standard deviation, kurtosis, and skewness of *V*_*e*_ were significantly different between the two groups, *P* < 0.05. There were significant differences in the mean and standard deviation of *V*_*p*_ between the two groups, *P* < 0.05.

### 3.3. Comparison of Histogram Parameters of Different Grades of Glioma

**Figure 5:**
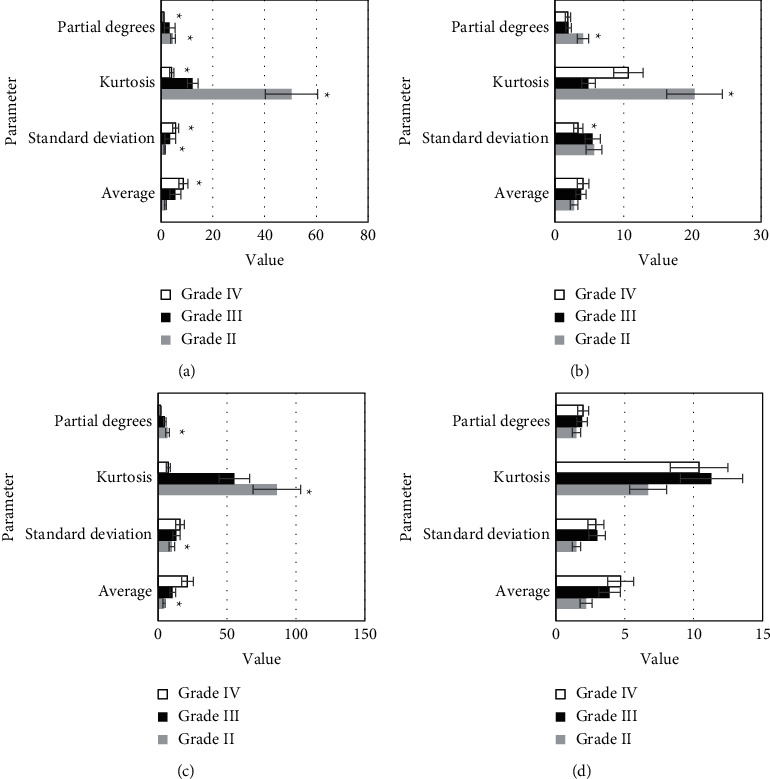
Comparison of histogram parameters of different grades of gliomas. (a) *K*^trans^; (b) *K*_*ep*_; (c) *V*_*e*_; (d) *V*_*p*_ compared with grade III glioma,  ^*∗*^*P* < 0.05.

The comparison results of the histogram parameters of different grades of glioma are shown in Figure 5. There were significant differences in *K*^trans^ standard deviation, kurtosis, and skewness between grades II and III. *K*_*ep*_ kurtosis and skewness were significantly different. The mean values of *V*_*e*_ were significantly different with *P* < 0.05. There were significant differences in *K*^trans^ average, kurtosis, and skewness between grades III and IV gliomas. *K*_*ep*_ standard deviation was significantly different. There were significant differences in mean value, standard deviation, kurtosis, and skewness of *V*_*e*_, all *P* < 0.05.

### 3.4. Receiver Operating Characteristic (ROC) Analysis of *K*^trans^, *K*_*ep*_, *V*_*e*_, and *V*_*p*_ Histogram Parameters in Glioma Grading

**Figure 6:**
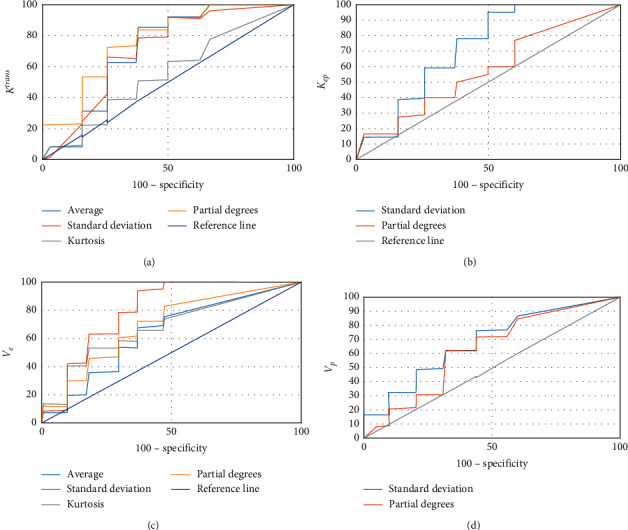
ROC curves of each parameter for diagnosing glioma. (a) *K*^trans^; (b) *K*_*ep*_; (c) *V*_*e*_; (d) *V*_*p*_.

Table 1 and Figure 6 show the results of the efficacy analysis of *K*^*trans*^, *K*_*ep*_, *V*_*e*_, and *V*_*p*_ histogram parameters for diagnosing glioma. The histogram parameters of *K*^trans^, *K*_*ep*_, *V*_*e*_, and *V*_*p*_ all showed good performance in the diagnosis of glioma, especially the *K*^trans^ value had the best performance, and its standard deviation had the best diagnostic performance among all parameters.

### 3.5. Correlation between Ki-67 Index and Various Parameters in HGG and LGG

**Figure 7:**
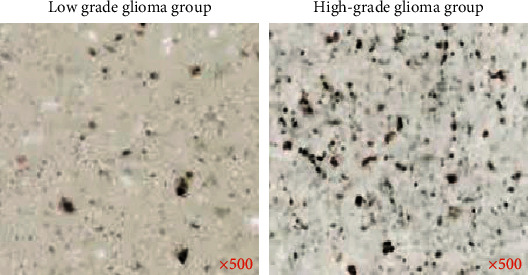
Comparison of Ki-67 index between HGG and LGG.

**Figure 8:**
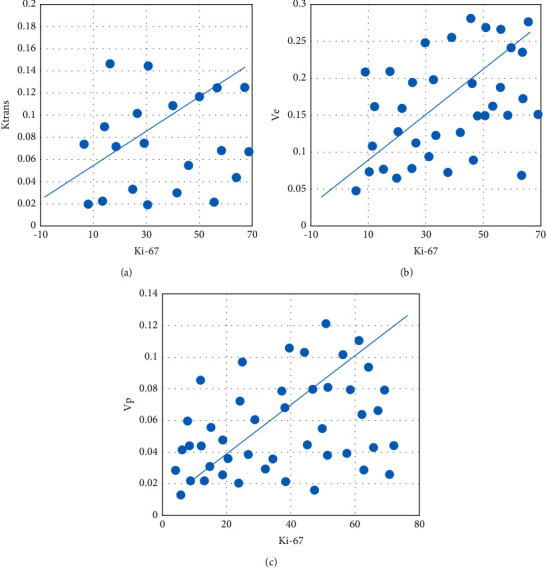
Correlation comparison between Ki-67 index and various parameters in HGG and LGG. (a) *K*^trans^; (b) *V*_*e*_; (c) *V*_*p*_.

Figures 7 and 8 show the comparison results of the correlation between the Ki-67 index of HGG and LGG and various parameters, and the results of Ki-67 immunohistochemical staining. Analysis of Figure 7 shows that the Ki-67 index of patients with HGG was higher than that of patients with LGG. Analysis of Figure 8 shows that *K*^trans^, *V*_*e*_, and *V*_*p*_ were positively correlated with Ki-67, and *K*_*ep*_ had no correlation with Ki-67.

## 4. Discussion

Glioma is the most common intracranial primary tumor, which is characterized by high vascularization, high heterogeneity, and strong invasiveness. In recent years, despite the continuous advancement of surgical and chemotherapy techniques, the survival of glioma patients after comprehensive treatment is still very low [16]. Clinical studies showed that the survival of patients with gliomas will not exceed two years after diagnosis, and almost all LGG will develop into HGG [17]. The incidence of glioma has also shown an upward trend year by year. Glioma has high morbidity and mortality, which poses a huge threat to human life safety [18]. The diagnosis and grading of gliomas in the early stages of the disease have an important impact on the determination of the disease treatment plan and the prognosis of the disease. Many clinical studies suggested that microvascular proliferation is an important histological characteristic of gliomas in the brain, and the degree of microvascular proliferation also increases with the increase of tumor grade. Compared with normal blood vessels, tumor neovascularization is immature and its permeability is high, so it is easy to cause leakage of intravascular contrast agent. The above changes are one of the important indicators for the diagnosis of glioma [19].

In recent years, with the continuous progress and development of imaging technology, many new technologies have been developed in the diagnosis of craniocerebral diseases. DCE-MRI is a novel functional MR imaging technique based on microvascular permeability and pharmacokinetic model assumptions. Based on the magnetic field changes caused by the leakage of contrast agent, it can quantitatively measure the microvascular permeability of gliomas and then noninvasively, dynamically, and quantitatively evaluate the functional properties of microvessels and improve the accuracy of glioma grading [20–22]. Quantitative parameters derived from the DCE-MRI hemodynamic model include the *K*^trans^, *K*_*ep*_, *V*_*e*_, and *V*_*p*_. In recent years, DCE-MRI has been widely used in the differential diagnosis of single brain metastases, primary central nervous system lymphoma (PCNSL), and glioma [23]. At present, the results are not uniform for the selection of optimal parameters and optimal thresholds for DCE-MRI in glioma grading. The reason may be that the quantitative parameters and arterial input function (AIF) are obtained in different ways in different studies, and the number of study cases will also have a certain impact on the results [24]. Therefore, the application value of DCE-MRI in the diagnosis of glioma needs further exploration and in-depth research. In this study, patients with glioma were diagnosed by DCE-MRI. The results showed that the mean, standard deviation, kurtosis, and skewness of *K*^trans^ and *V*_*e*_, standard deviation and skewness of *K*_*ep*_, and mean and standard deviation of *V*_*p*_ were statistically significant in differentiating HGG from LGG (*P* < 0.05). ROC analysis showed that the above values had good diagnostic performance for differentiating HGG from LGG, and *K*^trans^ had the highest standard deviation diagnostic accuracy. The standard deviation, kurtosis, and skewness of *K*^trans^, kurtosis and skewness of *K*_*ep*_, and mean value of *V*_*e*_ were statistically significant in differentiating grade II and III gliomas (*P* < 0.05). The mean, standard deviation, kurtosis, skewness of *K*^trans^ and *V*_*e*_, and standard deviation of *K*_*ep*_ were statistically significant in differentiating grade III and IV gliomas (*P* < 0.05). *K*^trans^, *V*_*e*_, and *V*_*p*_ were positively correlated with Ki-67. This indicates that DCE-MRI histogram-related indicators are of great significance in the diagnosis and grading of glioma. This can provide important reference information for clinical treatment. This is consistent with the results of some previous studies.

In addition to grading gliomas after diagnosis, how to segment tumors from MRI images is also a focus and a difficulty in clinical research. At present, clinical segmentation of glioma tumor regions mainly relies on manual selection and division by doctors. This segmentation method is not only time-consuming and labor-intensive, but also the segmentation results are greatly influenced by the doctor's subjective opinion and require the doctor to have a strong professional knowledge reserve [25, 26]. This is a huge waste of time, manpower, and material resources. Therefore, it is necessary to develop an automatic tumor segmentation system. In recent years, deep CNN models have received extensive attention and applications in the field of medical image segmentation [27]. Abundant related research results showed that the CNN-based algorithm can directly use the original image as input, and automatically extract the characteristics of image features. It presented considerable advantages compared with traditional segmentation algorithms in the field of computer vision [28, 29]. At present, scientists have also done a lot of research on its application in glioma segmentation. For example, some scholars proposed a complex two-channel CNN model and applied it to the segmentation of glioma [30]. In this work, the CNN algorithm was applied to the segmentation of DCE-MRI images of glioma patients. The research results showed that CNN can better segment glioma lesions from the perspective of visual observation. In objective indexes, the CNN algorithm performed better than traditional segmentation algorithms on quantitative indexes such as MSE, PSNR, and SSIM. This shows that the CNN algorithm has high application value in DCE-MRI image segmentation.

## 5. Conclusion

Patients with glioma were taken as research objects, and MRI with the CNN algorithm was used to classify glioma and segment glioma. The results showed that the CNN algorithm had good performance in DCE-MRI image segmentation of glioma patients. The DCE-MRI histogram of glioma of different grades showed significant differences in *K*^trans^, *K*_*ep*_, *V*_*e*_, and *V*_*p*_, and other indicators, and the correlation between *K*^trans^ , *K*_*ep*_, *V*_*e*_, and *V*_*p*_, and other indicators in Ki-67 also showed significant differences. In summary, CNN and DCE-MRI have high clinical application value in the diagnosis and differentiation of glioma. Nevertheless, there are still some defects in this work. For example, this research only analyzed the effect of DCE-MRI on the diagnosis of glioma and did not compare it with other diagnostic methods, which did not prove that DCE-MR is the best method for the diagnosis of glioma. In future studies and work, we will improve the above problems and further address them.

## Figures and Tables

**Table 1:** The results of analysis of the efficacy of *K*^trans^, *K*_*ep*_, *V*_*e*_, and *V*_*p*_ histogram parameters in diagnosing glioma.

		95% confidence interval (CI)	Sensitivity (%)	Specificity (%)
*K* ^trans^	Mean	0.88 (0.65,0.99)	88.6	85.1
	Standard deviation	0.88 (0.65,0.99)	84.2	85.7
	Kurtosis	0.88 (0.65,0.99)	93.8	77.7
	Skewness	0.88 (0.65,0.99)	95.5	77.7

*K*_*ep*_	Standard deviation	0.88 (0.65,0.99)	44.3	100
	Skewness	0.81 (0.77,0.90)	88.1	86.2

*V*_*e*_	Mean	0.83 (0.72,0.96)	83.9	94.1
	Standard deviation	0.78 (0.71,0.89)	77.2	68.8
	Kurtosis	0.85 (0.69,0.90)	74.4	85.5
	Skewness	0.83 (0.78,0.94)	79.8	86.7

*V*_*p*_	Mean	0.88 (0.65,0.99)	88.2	77.3
	Standard deviation	0.77 (0.63,0.92)	95.5	70.1

## Data Availability

The data used to support the findings of this study are available from the corresponding author upon request.
